# Thermoelasticity of Injection-Molded Parts

**DOI:** 10.3390/polym15132841

**Published:** 2023-06-27

**Authors:** Janez Turk, Daniel Svenšek

**Affiliations:** 1 Department of Physics, Faculty of Mathematics and Physics, University of Ljubljana, SI-1000 Ljubljana, Slovenia; daniel.svensek@fmf.uni-lj.si; 2 HELLA Saturnus Slovenija d.o.o., Letališka cesta 17, SI-1000 Ljubljana, Slovenia; 3 Laboratory of Molecular Modeling, National Institute of Chemistry, SI-1001 Ljubljana, Slovenia

**Keywords:** sink mark, volumetric shrinkage, injection molding defect

## Abstract

In the realm of injection-molded parts, small length scale deformation defects such as sink marks often pose a major challenge to the aesthetics or functionality of the parts. To address this problem, we present a comprehensive thermoelastomechanical approach that calculates the deformation of injection molded plastic by solving the elastic problem at each time step. In our methodology, two treatments of the molten core are considered: one as a liquid and the other as a rubbery state. Our results suggest that the rubbery state treatment provides higher accuracy in predicting the deformation results, as it maintains the displacement of the localized thermal shrinkage in its vicinity. The validity of our method is supported by empirical measurements on produced parts from the existing literature as well as on samples that we molded independently.

## 1. Introduction

Injection molding of thermoplastic polymers is a widely used production process in which molten plastic is injected at high temperature into a metal cavity and cooled until it solidifies into a functional part. This process is essential to the mass production industry due to its scalability and low unit price, which keeps production costs manageable. Considering the low profit margins and high capital intensity associated with acquiring steel molds for plastic injection molding, it is critical to avoid costly mold and part design mistakes. Broken molds or visually deficient parts can have a devastating impact on profitability, which has led to the emergence of slogans such as “right on the first try”. To consistently achieve such production goals, simulation of the injection molding process is required. This involves solving the relevant partial differential equations for a given part geometry, considering appropriate boundary conditions as well as material models, and generating results that predict part moldability and quality. The prediction of warpage, i.e., the deviation of the shape of the produced part from the intended shape, is the pinnacle of modern simulation software [1,2,3].

**Figure 1:**
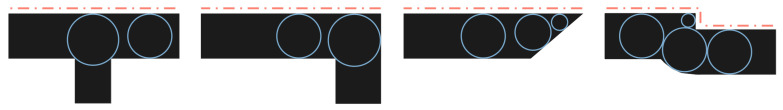
Various typical cross sections of plastic parts, with the differences in thickness illustrated by the different sizes of the maximum spheres. Surfaces where visual quality is vital and where deformation is of particular concern are marked by dashed lines.

In addition to warpage, there are many other defects that can occur in injection molded parts, and the goal of simulation is to predict as many defects as possible. One particular defect that occurs in injection molding is a “sink mark”, an undesirable depression on the surface of the part caused by localized high shrinkage of the material. Typically, sink marks occur in areas of the part that are thicker and cool more slowly, causing the surface to shrink away from the cavity wall more than adjacent areas. The local thickness is best defined as the diameter of a maximum sphere contained locally in the part, as shown in Figure 1 [4].

The aim of this work is to show that both global warpage and local depressions are due to the same cause, namely, thermal contraction during cooling and solidification of the plastic under pressure. Although this interpretation has been suspected in the literature, it has not yet been clearly established. The exact elastomechanics of the solidified layer in at least two dimensions throughout the production cycle must be calculated to show that sink marks and warpage are the same deformation manifested at different length scales. Furthermore, this work aims to broaden the definition of “sink marks” to include both depressions under a typical rib, as shown on the far left in Figure 1, and local surface deformations caused by any local variation of thickness.

### 1.1. Historical Overview of Injection Molding Process Modeling

An excellent historical overview of the academic field of injection molding simulation is provided by Peters Kennedy’s book [5], which serves as a common entry point for this field of study. The beginnings date back to 1960 when electronic computers were introduced and the first non-isothermal calculation of polymer flow in a simple rectangular plate cavity was performed [6]. This calculation involved modeling the increase in viscosity due to heat loss and discussing the resulting influence on the molding process. It then took 18 years of academic demonstrations before the first commercial company, Moldflow, was founded by Colin Austin in 1978. Engineers now had access to academic-grade code and the book Moldflow Design Principles [7], which still serves as the benchmark for the field. At that time, numerical techniques were used to reduce three-dimensional sheet-like geometries to equivalent flow in a plane [8].

With the increasing popularity of computer-aided design systems (CAD) in the 1980s, midplane shell models emerged. It became possible to solve the relevant equations using the finite difference method over the thickness of the part and to propagate them over the entire component using the finite element method [9]. It should be noted, however, that this type of modeling cannot capture the local variation in thickness. Academic work paved the way for modeling the packing phase, i.e., the time after the cavity is completely filled but the plastic is still molten and under pressure [10,11,12,13]. During this period, commercial companies began to develop the calculation of heat transfer both through the mold and through warpage. Moldflow led the way in warpage prediction, with a phenomenological approach, molding and simulating 28 sample plates with different parameters. The shrinkage of the sample plates was measured and used to calibrate the parameters of the “Residual Strain” model [14].

In the 1990s, CAD systems evolved to produce three-dimensional models. Full three-dimensional simulation using the “volume of fluid” method gained traction [15], but computer power remained limited and workarounds were developed. The most renowned approach is arguably dual domain technology [16], where the three-dimensional CAD geometry surface is meshed and computational cells are formed from matching mesh elements on the opposite surfaces of the part. As computing power increased, full three-dimensional simulation became viable [17], leading to a major expansion of simulation functionality. Increased accuracy enabled direct calculation of phenomena such as warpage [18] and three-dimensional velocity fields.

Open source software for injection molding simulation was virtually nonexistent until the early stages of development were begun in [19]. The resulting hydrodynamic flow simulation code [19] is based on a solver within the popular open source finite volume library OpenFOAM [20]. Over time, functionalities for the calculation of fiber orientation and crystallization were added [21]. Validation was performed [22] and good performance was shown.

### 1.2. Modeling the Deformation of Injection Molded Parts

During the injection molding process, the plastic is exposed to significant temperature and pressure changes as the phase boundary between solid and molten plastic progresses. Already-solidified layers prevent newly formed layers from freely changing shape, as they are all subject to elastomechanics as an integral domain. Early attempts to model shrinkage of injection molded parts relied only on thermodynamic (pvT) characterization of density [23] to predict both isotropic stress and shrinkage. Later, a line of research was devoted to viscoelastic formulations of the problem, which is complex from both theoretical and experimental point of view, as it is difficult to measure viscoelastic material properties in a satisfactory way [24]. Nevertheless, Douven [25] has shown that the stresses calculated by elastic and viscoelastic descriptions are similar. Moreover, it was shown in parallel by Baaijens [26] that the flow-induced stresses are two orders of magnitude smaller than the thermal and pressure-induced stresses. Therefore, the elastic thermomechanical approach proved sufficient, which provided the basis for its validity for a group of authors who worked on its increasingly rigorous formulation [27,28]. These efforts culminated in a paper by Jansen [29] in which a fully elastic thermomechanical method for calculating residual stress and shrinkage was developed. Their method showed that the boundary conditions at the interface between the plastic and the mold play a more decisive role than the increasingly complex material characterization. The objective of the present study is to take up Jansen’s approach and adapt it for a two-dimensional cross-section of an injection molded part. Specifically, the aim is to predict the deformation of the part surface with a characteristic length scale of the part thickness.

### 1.3. Modeling the Formation of Sink Marks

Sink marks were first discussed by Marchewka in 1974 [30]. In their study, plates with ribs were molded under different processing conditions and sink marks were visually assessed. The study focused primarily on the visibility of sink marks as a function of rib thickness, distance from the gate, and surface finish of the affected area. The results of the study led to the establishment of a design guideline that a rib thickness of less than 60% of the base plate should not produce visible sink marks under most processing conditions. In addition, the study found that the distance from the gate is an important factor affecting the visibility of sink marks.

Later, Naka et al. [31] of what is now Phillips Corporation developed a method that used finite differences to calculate the evolving temperature distribution in a two-dimensional part cross section during injection molding. The temperature change was used as a thermal load for one-dimensional fiber elements that were independent of each other and constrained in the longitudinal direction. Only after the pressure in the cavity reached atmospheric values were the thermal, plastic, and elastic strains calculated over time and finally converted into displacements in the cross-sectional plane using the Poisson effect for the elastic strains. The numerical predictions of the method were compared with plates molded with an experimental tool where different values for rib thickness, rib height, and base plate thickness could be set. In addition, the mold was equipped with a pressure transducer to ensure that the pressure curve was known, although simulation of the packing phase was not yet generally available. The plates were molded with a profile measuring device and good agreement with the simulation was demonstrated.

In the late 1980s and early 1990s several influential doctoral studies were carried out in the Department of Mechanical Engineering at Ohio State University. The first thesis by Ramachandra [32] involved an extensive experimental investigation of sink mark depth on a test plate with varying rib thickness and rib root radius, as well as an investigation of the effects of process parameters such as melt temperature, mold temperature, and packing pressure. The results of the study have been cited in numerous subsequent studies, and have even found their way into the calibration report of a commercial software [33]. The second thesis by Reifschneider [34] formulated a finite element approach to calculate thermal shrinkage after the pressure in the cavity had dropped to zero and the plastic was able to separate from the mold walls. This work included a comprehensive discussion of the boundary conditions of the displacement field. Finally, Beiter [35] developed a geometric index to predict the depth of sink marks for a given packing pressure. These three theses represent important contributions to the understanding of sink mark formation in injection molding.

The research was continued by Mahesh Gupta of Michigan Technological University, who used the commercial software C-Mold [36] to simulate the injection molding process for the samples produced and measured by Ramachandra [32]. The researchers transferred the temperature results calculated by C-Mold to their own two-dimensional computational domain and continued calculating until a homogeneous temperature was reached. In this way, they ensured that all details from the filling phase and flow effects were taken into account. When the pressure of the C-Mold simulation dropped to zero, a structural analysis was carried out to calculate the thermal shrinkage. The material was assumed to be elastic-perfectly plastic, with temperature-dependent elastic modulus and yield stress around the glass transition temperature. The results were compared with measurements from Ramachandra [32] and Beiter [35]. In the next article by Shi and Ramachandra [37], the study was extended to analyze the necessary size of the computational domain to obtain the same results as in the full part analysis along with the influence of the mesh size. A year later, a three-dimensional analysis was introduced in the same way [38], and the plate used in Beiter’s study [35] was analyzed with two ribs crossing in the middle.

Subsequently, several experimental studies were conducted using methods such as design-of-experiment and genetic algorithms [39,40,41,42]. New modifications of conventional injection molding techniques have been explored, including rapid heating and cooling [43], gas-assisted injection molding [44], and microcellular injection molding [45]. An interesting departure from these studies is the use of artificial intelligence trained on a large dataset of process parameters and quality assessment results [46]. Finally, in the area of numerical sink mark prediction, one study [47] considered complex elastic material properties and a cooling rate-dependent equation of state for semi-crystalline plastic, but predicted only one-third of the measured sink mark depth.

All the studies mentioned above start by calculating the deformation of the solidifying plastic when the pressure at the point of interest reaches the atmospheric value and show that such an approach can correctly predict the depth of the sink marks. However, by disregarding the exact pressure history of the filling and packing phases, the large-scale warpage is conceptually decoupled from the small-scale deformations of the parts. Moreover, all of these studies employ fairly complex elastomechanical material models such as elastic-perfectly plastic [31,36,37,38], thermo-viscoplastic [34], or complex temperature- and strain rate-dependent [47] models. In this paper, we investigate the predictive power of a method (which we refer to as the “thermoelastic method”) that uses data available in material characterization reports of a commercial injection molding simulation software, treating the solid phase as an elastic material and the molten phase as either a pure liquid or a rubbery material with an elastic modulus reduced by three orders of magnitude.

## 2. Thermoelastic Method

**Figure 2:**
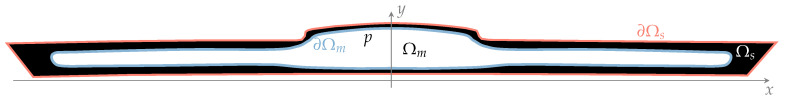
Computational domain Ω at a given time, divided into solidified ($\Omega_{s}$) and molten ($\Omega_{m}$) subdomains. They are characterized by different physical properties and change with time due to the time-dependent phase boundary $\partial \Omega_{m}$. The outer boundary $\partial \Omega_{s}$ is fixed.

We have developed a method for predicting the local deformation of injection molded parts by dynamically calculating the displacement and stress fields of the solidifying plastic. Plastic parts are typically sheet-like, which means that some of their geometrical features can be adequately represented numerically by a two-dimensional cross-section, as already shown in Figure 1. A two-dimensional computational domain Ω in the shape of the mold cavity is defined in the xy-plane, as shown in Figure 2. For simplicity, we assume an infinite dimension in the direction of the *z*-axis. We assume that plastic flow occurs only in the direction of the *z*-axis, so that the cross section under study is filled instantaneously at time zero. This assumption eliminates the need to model the flow. Flow-induced residual stresses are not modeled in this study. As established by Baaijens [26], they are two orders of magnitude smaller compared to thermal and pressure-induced stresses.

As soon as the cross-section under investigation is filled with hot plastic melt, heat transfer to the colder mold with homogeneous temperature begins, initiating the evolution of the temperature field of the plastic T(x,y,t). It is in the nature of the injection molding process that the plastic part cools throughout the cycle, resulting in the continuous growth of a layer of solidified plastic cooled below the glass transition temperature $T_{g}$. Accordingly, we dynamically divide the computational domain Ω into time-dependent solidified $\Omega_{s}$ and molten $\Omega_{m}$ subdomains with a time-dependent phase boundary $\partial \Omega_{m}$ and fixed outer boundary $\partial \Omega_{s}$, as shown in Figure 2. The solidified domain $\Omega_{s}$ is treated as an elastic solid in all phases.

**Figure 3:**
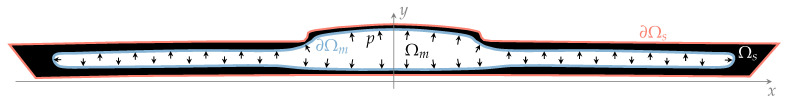
The computational domain at a given time in the filling and packing phase, as well as in the cooling phase treated with the liquid approach. In these phases, the elastic problem is solved only in the solidified domain $\Omega_{s}$. The arrows represent the homogeneous pressure force of the liquid melt core exerted on the phase boundary.

During the filling and packing phases, a hydrodynamic plastic flow in the *z*-direction exists in the molten $\Omega_{m}$ subdomain, which is controlled externally by the volumetric filling rate or the packing pressure. The hydrodynamic pressure p(x,y,z,t) corresponding to this flow is provided by the commercial software Autodesk Moldflow 2023. As verified, this pressure field can be considered homogeneous in the cross-sectional plane to a good approximation. Thus, the time-dependent pressure field p(z,t) evaluated at the center of the investigated cross-section serves as a boundary condition at the phase boundary $\partial \Omega_{m}$, as shown in Figure 3.

In the packing phase, the flow through a cross-section compensates for the thermal shrinkage of the downstream plastic. For a given cross-section, the packing phase ends when the hydrodynamic pressure at that cross-section drops to zero (the ambient pressure of the vented mold). This occurs when the packing flow at the set packing pressure can no longer accommodate the downstream thermal shrinkage due to the large cooling-induced viscosity increase of the upstream plastic. At this point, thermal shrinkage prevails and the pressure at this cross-section begins to drop. When it reaches the ambient value, the pressure gradient in the *z*-direction disappears and the flow through this cross-section is completely stopped.

In the final hydrostatic cooling phase, the pressure connection to the molding machine is interrupted and there is no longer any transport of the melt in the *z*-direction. The hydrodynamically isolated molten core continues to shrink thermally together with the solidified plastic, while there is no pressure that would prevent the solidified plastic from detaching from the mold walls.

**Figure 4:**
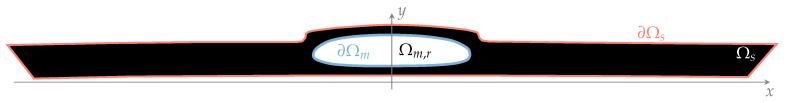
The computational domain at a given time in the cooling phase treated with the rubber approach. The elastic problem is solved in the whole domain. The plastic in the molten domain $\Omega_{m,r}$ is in a rubbery state characterized by a shear modulus reduced by three orders of magnitude.

In this phase, we treat the molten region $\Omega_{m}$ with two approaches, either as a liquid with a shear modulus of zero as before in Figure 3, or as a rubbery material with a finite shear modulus that is three orders of magnitude lower compared to the solidified plastic, as suggested in previous studies [36,48,49] (Figure 4). In the latter case, the complete elastic problem is solved in the entire domain. In the former case, the change Δp of the uniform pressure in the liquid core is obtained by equating the change in volume of the liquid due to thermal shrinkage and compression/dilation with the change in the volume of the cavity due to elastic displacement Δu of the solidified plastic at the $\partial \Omega_{m}$ boundary and solving for Δp by numerical iteration:

(1) $$\int_{\Omega_{m}}\;\;\;dS\;\alpha_{m}\left(r\right)\Delta T\left(r\right)-\Delta p\int_{\Omega_{m}}\;\;\;dS\;K_{m}^{-1}\left(r\right)=\oint_{\partial \Omega_{m}}\;\;\;dl\;\hat{n}\;\cdot \;\Delta u\left(\Delta T,\Delta p\right).$$

In the surface integrals on the left, $\alpha_{m}$ and $K_{m}$, are the coefficients of thermal expansion and the bulk modulus of the liquid melt (Appendix A); r=(x,y). In the edge integral on the right, $\hat{n}$ is the outward normal of the $\Omega_{m}$ domain and Δu corresponds to a particular field ΔT(x,y) in the solidified domain and to the sought Δp in the liquid.

The plastic, already solidified at a given time, undergoes elastic deformation caused by the changes in the time-dependent stress field due to thermal shrinkage and/or the changes in the time-dependent pressure p(t) of the molten core. As solidification progresses, the solidified and molten regions constantly change. Therefore, at each time *t* we are dealing with a *separate* elastic problem defined only during a short time interval Δt for the region $\Omega_{s}\left(t\right)$, which is solid at time *t*. Similarly, in the rubbery core treatment of the cooling phase we are dealing with an elastic problem defined only in the time interval Δt for the regions $\Omega_{s}\left(t\right)$ and $\Omega_{m,r}\left(t\right)$ as they occur at time *t*.

The result of the elastic problem in the time interval Δt is an increment $\Delta u_{i}$ of the displacement field and an increment $\Delta \sigma_{ij}$ of the stress field, with which the total displacement field $u_{i}\left(t\right)$ and the stress field $\sigma_{ij}\left(t\right)$ are updated:

(2) $$\sigma_{ij}\left(t\right)=\sum^{t}\Delta \sigma_{ij},\;u_{i}\left(t\right)=\sum^{t}\Delta u_{i}.$$

The stress field describes the physical state of the material, and its divergence is zero at all times. In contrast, the total displacement field relates directly to the physical strain only in the regions not traversed by the $\partial \Omega_{m}$ phase boundary during evolution. Strictly speaking, in our case this is only the outer boundary. However, in the case of overmolding, for example, where cool plastic is placed in the larger mold cavity and then molded over, this “regular” region would be larger.

The crucial implication is that both the final stress state and the final shape of the cross-section generally depend on the specific time course of all relevant variables, i.e., of the fields T(x,y,t) and p(t), and not on the mere difference between their final and initial values, as would be the case with a constant $\Omega_{s}$ and $\Omega_{m,r}$.

**Figure 5:**
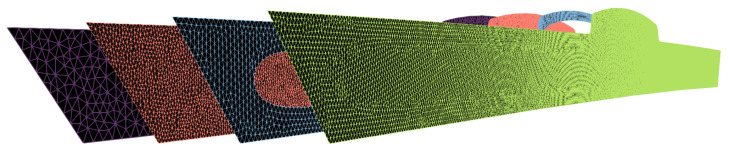
The heat equation solved on a coarse mesh (purple). A mesh for the thermoelastic problem is generated using the resulting temperature field. When the molten core is treated as a liquid, only the solidified subdomain $\Omega_{s}$ is meshed (blue); conversely, when treated as a rubbery material, the entire domain is meshed (green), ensuring that the element edges follow the phase boundary $\partial \Omega_{m,r}$. Subsequently, the solutions of the thermoelastic problem are mapped onto a finer mesh (red), where they are accumulated over time steps according to Equation (2).

The heat equation (Section 2.1) and the thermoelastic problem (Section 2.2) are solved numerically with the finite element method [50] using a suitable discretization; see Figure 5. The discretization of the elastic domains $\Omega_{s}$ and $\Omega_{m,r}$ is updated at each time step according to the change in the shape of the phase boundary $\partial \Omega_{m}$. The increments of the displacement and stress fields from the individual time steps of the elastic problems are accumulated by mapping them onto a mesh of the entire domain Ω, which must be fine enough to capture the details of the displacement and stress field increments of all time steps. The heat equation, on the other hand, allows for a coarser mesh of Ω, which is used in this case.

### 2.1. Heat Equation

The evolution of the temperature field T(x,y,t) in the entire domain Ω is modeled by the heat equation

(3) $$\rho c_{p}\dot{T}=\nabla \;\cdot \;\left(\lambda \nabla T\right),$$

where $\dot{T}\equiv \partial T/\partial t$. We assume that there is no temperature gradient along *z*, and consequently no heat flux in this direction. The temperature-dependent density ρ, specific heat $c_{p}$, and thermal conductivity λ of the plastic material can be obtained from material characterization reports provided by commercial software for thousands of plastic grades. For certain materials, the thermal parameters λ and $c_{p}$ are measured over a wide temperature range, as discussed in Appendix B, while for others only a single value is provided. The density is determined by the pvT relation, as described in Appendix A, and is commonly available.

**Figure 6:**
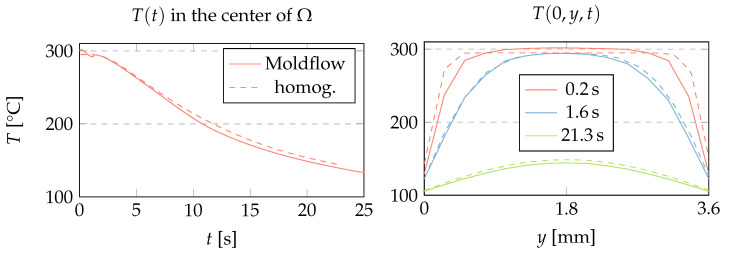
Comparison of temperature fields calculated by Moldflow and by Equation (3) with homogeneous initial temperature $T_{m}$ on Ω (dashed lines) and the boundary condition from Equation (4). The center of Ω refers to the point that solidifies last.

A homogeneous initial condition ${T\left(t=0\right)|}_{\Omega }=T_{m}$ is employed, where $T_{m}$ is the average temperature of the melt that has reached the investigated cross section. Typically, this value is a few degrees below the melt temperature, which is a process parameter. Such an average temperature result provided by commercial software takes into account the average viscous heating and conductive heat loss experienced by the plastic on its way to the investigated cross-section. In general, it would be ideal if our method had access to the entire temperature field calculated by detailed modeling of the filling phase. However, we argue that the homogeneous initial condition for the specific cross-section and process setup leads to a temperature field comparable to the Moldflow calculation (Figure 6).

Due to the imperfect contact between the polymer and the mold walls, the heat transfer through this interface must be modeled with a heat transfer coefficient *h* [51], which implies the boundary condition

(4) $$\lambda {\left.\nabla T\right|}_{\partial \Omega }\;\cdot \;\hat{n}=h\left(T_{\mathrm{mold}}-T_{\partial \Omega }\right),$$

where $\hat{n}$ is the outward normal of ∂Ω. While the appropriate value of *h* is widely discussed in the literature [51,52], we use the same approach as Moldflow [53] with specific values for the filling, packing, and cooling phases:

(5) $$h_{\mathrm{filling}}=5000\frac{W}{m^{2}K},\;h_{\mathrm{packing}}=2500\frac{W}{m^{2}K},\;h_{\mathrm{cooling}}=1250\frac{W}{m^{2}K}.$$

**Figure 7:**
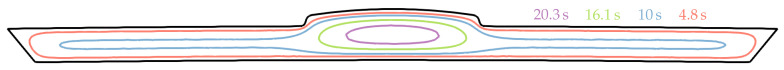
Phase boundary shown at different times. Areas with greater thickness take longer to cool.

Figure 7 illustrates the evolution of the phase boundary, which initially matches the shape of the computational domain and then gradually contracts to enclose regions with larger heat capacity that take longer to cool.

### 2.2. Elastomechanics

The solidified/rubbery plastic is treated as an elastic material obeying Hooke’s law [54] for changes in the strain $\Delta u_{ij}\left(x,y,t\right)$ and stress $\Delta \sigma_{ij}\left(x,y,t\right)$ fields in individual time steps Δt with well-defined computational domains $\Omega_{s}\left(t\right)$ and $\Omega_{m,r}\left(t\right)$:

(6) $$\Delta \sigma_{ij}=K\Delta u_{kk}\delta_{ij}+2G\left(\Delta u_{ij}-\frac{1}{3}\Delta u_{kk}\delta_{ij}\right)-K\alpha \Delta T\delta_{ij},$$

where *K* and *G* are the bulk and shear elastic moduli, respectively, and $\delta_{ij}$ is the Kronecker delta. The last term represents the stress due to thermal expansion/contraction, where α is the coefficient of thermal expansion. The temperature-dependent α and *K* are extracted from the pvT relation (Appendix A). The shear modulus is determined from *K* and the Young’s modulus *E*, which is measured at room temperature for the solid phase and is reduced by three orders of magnitude for the rubbery material, as discussed in Appendix C.

The displacement vector Δu(x,y,t) is calculated numerically by the finite element method using a standard weak (variational) formulation. Notwithstanding the fact that the initial stress $\sigma_{ij}\left(t\right)$ before the onset of Δu is nonzero, the elastic problem for Δu is as usual, because the initial state at time *t* is an equilibrium one. This can be seen immediately from the variation of the elastic free energy density

(7) $$\begin{array}{ll} \delta f & =\left(\sigma_{ij}+\Delta \sigma_{ij}\right)\delta \Delta u_{ij}=\left(\sigma_{ij}+\Delta \sigma_{ij}\right)\partial_{j}\delta \Delta u_{i} \end{array}$$

(8) $$\begin{array}{ll} & =\partial_{j}\left[\left(\sigma_{ij}+\Delta \sigma_{ij}\right)\delta \Delta u_{ij}\right]-\left(\partial_{j}\sigma_{ij}+\partial_{j}\Delta \sigma_{ij}\right)\delta \Delta u_{i}, \end{array}$$

where in line (7) we have used the fact that the stress tensor is symmetric. The first term of line (8) is a divergence, and is converted into a boundary contribution $\sigma_{ij}{\hat{n}}_{j}$ (with $\hat{n}$ the outward normal) which is balanced by external forces on the boundary, as the system is in equilibrium at time *t*. For the same reason, in the second term of line (8), $\partial_{j}\sigma_{ij}=0$ everywhere. Thus, the new equilibrium condition at time t+Δt simply requires $\partial_{j}\Delta \sigma_{ij}=0$, where $\Delta \sigma_{ij}$ is provided by Equation (6), which is the usual elastomechanical problem for Δu. From the first term of line (8), we obtain the boundary condition

(9) $$\Delta \sigma_{ij}{|}_{\partial }{\hat{n}}_{j}=\Delta \sigma_{ij}^{\mathrm{ext}}{\hat{n}}_{j},$$

where $\Delta \sigma_{ij}^{\mathrm{ext}}$ is any change in the external stresses on the boundary within the time interval Δt.

#### 2.2.1. Boundary Conditions

While the part is in the mold $\Delta u_{z}=0$ is assumed because of the shape of the part beyond the investigated cross-section that does not allow for shrinkage in the *z*-direction, simple ribs or a slight widening of the cross-section are sufficient to restrict the displacement along *z*. However, the elastic force is present in this direction, and the stress tensor has the form

(10) $$\Delta \sigma =\left[\begin{array}{ccc} \Delta \sigma_{xx} & \Delta \sigma_{xy} & 0\\ \Delta \sigma_{xy} & \Delta \sigma_{yy} & 0\\ 0 & 0 & \Delta \sigma_{zz} \end{array}\right].$$

**Figure 8:**
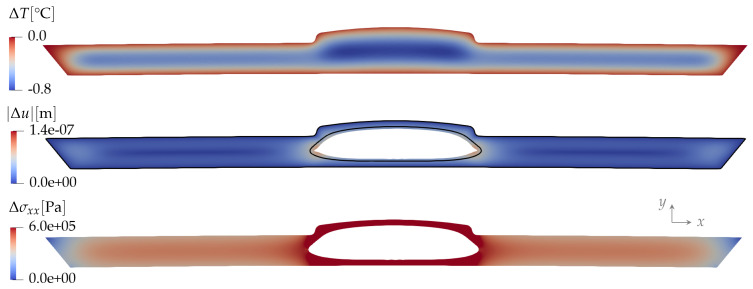
Fully constrained displacement at the outer boundary: magnitude of the displacement field |Δu| (**middle**) and the component $\Delta \sigma_{xx}$ of the stress field (**bottom**) resulting from a change in the temperature field ΔT(x,y) (**top**) and the pressure Δp=−0.5 MPa in the liquid core.

**Figure 9:**
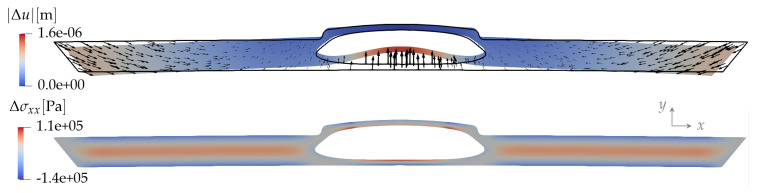
Free outer boundary: magnitude of the displacement field |Δu| (**top**) and the component $\Delta \sigma_{xx}$ of the stress field (**bottom**) resulting from the change in the temperature field (as in Figure 8) and the pressure Δp=−300 MPa in the liquid core. The color plot of the displacement is deformed by the displacement vectors enlarged by a factor of 1000.

On the outer boundary $\partial \Omega_{s}$, we use two types of boundary conditions:

- Fully constrained, meaning that while the plastic pushes against the mold, the displacement there is zero: ${\Delta u|}_{\partial \Omega_{s}}=0$. Consequently, the displacement is small everywhere, and most of the induced stresses cannot be relaxed. An example of the displacement and stress fields for this case is shown in Figure 8.
- Free boundary, meaning that when the contact force between the plastic and the mold reaches zero the plastic is free to detach from the mold. The boundary condition is now a zero force on the surface of the plastic, $\Delta \sigma_{ij}{\hat{n}}_{j}{|}_{\partial \Omega_{s}}=0$. The in-plane displacement allowed here relaxes most of the in-plane stress. The stress component $\sigma_{zz}$ cannot relax significantly because of the remaining constraint $\Delta u_{z}=0$. An example of the displacement and stress fields for this case is shown in Figure 9.

The actual boundary condition at the hard wall of the mold is complex, and may involve a mixture of the two cases or even a third scenario where the plastic is pressed against the mold wall but does not adhere to it, allowing tangential movement. Solidified plastic, however, usually moves away from the mold walls due to negative thermal shrinkage. Only a highly inhomogeneous thermal load could cause such global deformation that the plastic would push locally against the mold walls, as shown in Figure 9. However, limiting the local intrusion into the mold only affects the large-scale deformation of the part and not its local features such as sink marks, which are our main focus.

#### 2.2.2. Thermal Stress Relaxability: The Role of Geometry and Melt Elasticity

In the case of the free outer boundary, much of the thermally induced stress is relaxed by the displacement. The remainder consists of residual stresses due to the inhomogeneity of the thermal load and the elastic response of the medium, which together prevent complete relaxation in the presence of general thermal inhomogeneity.

**Figure 10:**
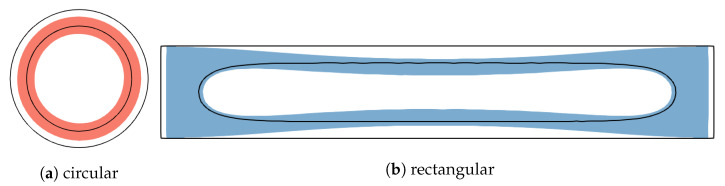
Deformation (colored) of partially solidified cross-sections with qualitatively different shapes and free outer boundary condition due to inhomogeneous thermal load in the cooling phase. The rectangular cross-section displaces a larger relative volume at the phase boundary compared to the circular cross-section, resulting in a much smaller pressure drop in the liquid core.

**Figure A3:**
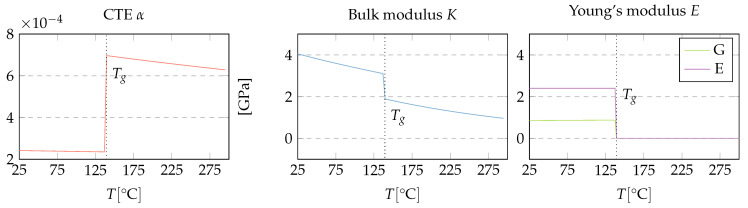
The coefficient of thermal expansion α and the bulk modulus *K* are calculated directly from the equation of state in Appendix A. The Young’s modulus *E* for the solid phase is measured at room temperature, and the value for the rubbery phase is three orders of magnitude lower. The shear modulus *G* is shown as well.

To demonstrate differences in this behavior, two cross-sections with different tendencies to displace the volume (Equation (1)) enclosed by the phase boundary during thermal shrinkage were studied:

- The circular cross-section (Figure 10a) displaces a minimal volume of solidified plastic at the phase boundary. Despite the (radial) inhomogeneities in the ΔT field, the displacement does not change the shape of the cavity, and it only shrinks according to the average effect of the temperature change. It should be noted that the coefficient of thermal expansion of a typical plastic in the molten state is about three times higher than that of the solid state; see Figure A3. Consequently, the equilibrium pressure resulting from the balance between the stiffness of the outer shell and the thermal shrinkage of the molten core (Equation (1)) is strongly negative.
- The rectangular cross-section in Figure 10b deforms in a more complex and global way. The inward bending of the thin long walls of solidified plastic displaces even more volume than the free thermal shrinkage of the molten core, resulting in a slightly positive equilibrium pressure in the core. Due to the low bending stiffness of the outer shell, a small pressure is sufficient to bring the displaced volume to an equilibrium magnitude.

**Figure 11:**
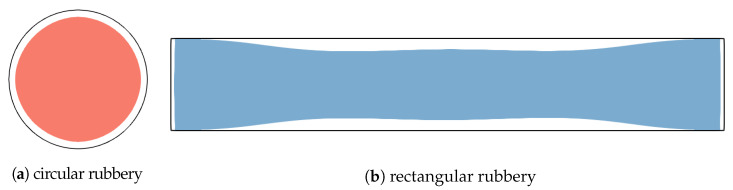
The same examples from Figure 10 except with a rubbery core with weak elasticity. The elongated geometry in (**b**), which is prone to deformation of the core cross section, is strongly affected by the nonzero elasticity of the melt (Figure 10b), while that in (**a**) is not.

The global bending deformation seen in Figure 10b is subject to global displacement of the core liquid; therefore, it depends crucially on its unobstructed flow. A finite, albeit very small, elastic modulus of the melt prevents its large-scale flow, which is required to compensate for global variations of the core cross-section. Consequently, global deformations affecting the shape of the cavity are suppressed in the rubbery case, as can be seen from the flattened bending deformation in Figure 11b. In contrast, geometries without such global deformations are not decisively affected by the weak elasticity of the melt; see Figure 11a.

**Figure 12:**
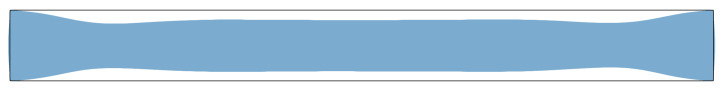
A sufficiently long rectangular domain exhibits homogeneous shrinking in the central region.

Figure 12 shows the result for an even longer rectangular domain. Such a cross-section corresponds to the simplest possible injection molded part, a plate. Empirically, it is clear that the homogeneous displacement in the center, away from localized end effects, corresponds to reality.

### 2.3. Ejection Warpage

When a part has cooled to a uniform temperature, it no longer deforms. As long as it remains in the mold with restricted displacement in the *z*-direction, it has a large $\sigma_{zz}$ component of the stress tensor. Other stress components may be significant if the displacement of the frozen layer has been constrained by the mold walls. When the part is removed from the constraints of the mold, the boundary conditions change to those of zero forces and a new elastic equilibrium must be determined in the ejection step.

**Figure 13:**
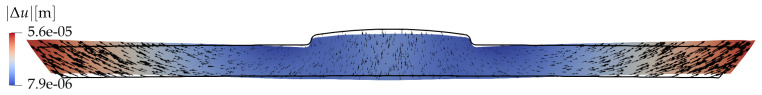
The displacement increment Δu in the ejection step; the vectors are magnified by a factor of 20. Shrinkage in the *z* direction implies expansion in the other directions due to the Poisson effect.

This is accomplished by solving another elastomechanical problem, analogous to the previous problems for each time step, with the boundary condition according to Equation (9) as before and by updating the displacement and stress fields according to Equation (2) as before. Thus, the boundary condition is now $\Delta \sigma_{ij}{{|}_{\partial }{\hat{n}}_{j}=-\sigma_{ij}|}_{\partial }{\hat{n}}_{j}$, where $\sigma_{ij}$ is the stress accumulated up to the event of ejection. In particular, $\Delta \sigma_{zz}=-\sigma_{zz}$, resulting in shrinkage along *z*. The calculated displacement field increment Δu (Figure 13) is added to the displacement field accumulated up to the ejection event to obtain the final total displacement field u of the plastic after ejection.

The deformation that occurs when a part is removed from the tool is commonly referred to as warpage. Commercial software usually calculates this type of deformation only once at the end of the production process. While the stress field required for this is not accumulated over the multitude of elastic problems of individual time steps, as in our thermoelastic method, it is an effective stress field constructed by various methods from simple to more sophisticated. One of the simplest is the linear shrinkage approach; this uses an isotropic stress field that, when released, causes the same local volumetric shrinkage as local cooling [18]:

(11) $$\sigma_{ij}\left(x,y\right)=K\frac{v\left(T\left(x,y\right)\right)-v\left(25\;{}^{\circ}C\right)}{v(T(x,y))}\delta_{ij}.$$

The local cooling shrinkage is calculated as the relative volume change between the local temperature T(x,y) at the beginning of the cooling phase and the reference room temperature 25 °C. The specific volume *v* is provided by the equation of state (Appendix A).

The present thermoelastic approach distinguishes between deformations inside the mold, which are subject to relevant boundary conditions, and those occurring outside the mold, where free boundary conditions apply. By applying the thermal load to the developing frozen layer rather than to the fully solidified cross-section, this method takes into account local intricacies, and thereby allows the prediction of deformation patterns with small characteristic length scales.

## 3. Results

The final surface deformation, which is the central result of the thermoelastic method, is now demonstrated using the example of a cross-section of an experimental specimen subjected to a time-dependent pressure p(t) at a packing pressure 50 MPa, which is examined in detail in Section 3.4.

**Figure 14:**
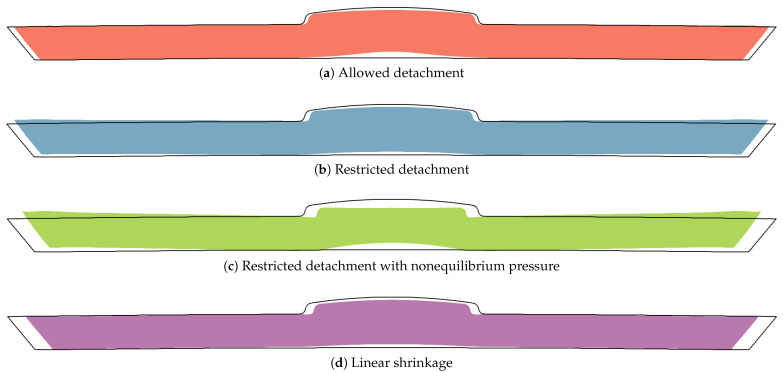
Comparison of final deformations scaled by a factor of 10, calculated using four approaches.

Figure 14 shows different deformations calculated with several variants of the method. In the region of increased thickness, a deformation in the form of a classical sink mark can be observed in all cases.

In the primary method shown in Figure 14a, the plastic is allowed to detach from the mold walls when the pressure drops.

In the simplified method shown in Figure 14b, detachment from the mold walls is restricted during all time steps. Instead of surface deformation, stress builds up, causing the surface of the part to deform only when it is removed from the mold. Although there is an equivalence between stress and displacement via Hooke’s law, this equivalence only applies to a single time step with an unchanged solidified domain. The stresses that are built up in earlier time steps when the solidified domain is thin and more deformable result in less displacement if it is not allowed at that time, but only later in a completely solidified domain. Consequently, smaller local deformation is expected when detachment is restricted, which is confirmed in Figure 14b.

In the ultimately simplified variant of the method shown in Figure 14d, the final deformation is calculated in a single step from the generally inhomogeneous isotropic stress field defined in Equation (11). This linear shrinkage approach yields a spread-out result that may predict global deformation reasonably well in the context of whole-part analysis, but fail to predict local defects. It can serve as a reference for the scope of an approach that solves only a single elastic problem.

The restricted detachment is interesting because it could be of similar accuracy to sophisticated residual stress prediction methods used by commercial software as an improvement to the isotropic shrinkage method [55]. If one wanted to limit the restricted detachment approach to a single calculation of the elastic problem, this would prevent determination of the actual instantaneous pressure in the molten core according to Equation (1). By neglecting the elastic displacement at the phase boundary, the pressure changes in the molten core are overestimated, while this is partially compensated by the underestimation of the local deformation due to the restriction of the detachment. The effect of such an approximation is demonstrated in Figure 14c. The sink mark depth is comparable to that in the primary method, but the surface opposite the sink mark deforms excessively, and the shape of the sink mark is different. Further investigation is needed to determine the validity of this approach.

### 3.1. Pressure of the Molten Core—Prediction of Voids

**Figure 15:**
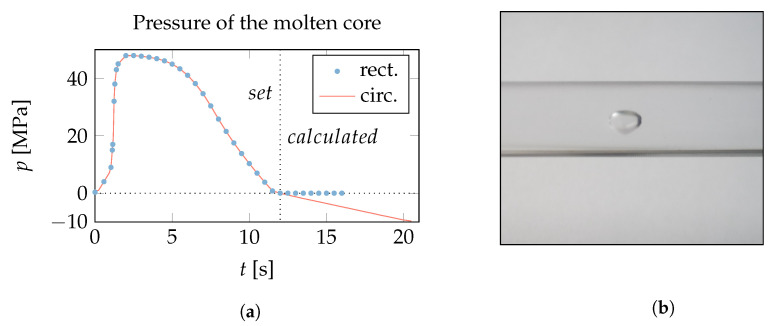
(**a**) Evolution of the pressure in the liquid molten core for the circular and rectangular shapes from Figure 10. The dotted vertical line marks the beginning of the cooling phase. In the circular case, a strong negative pressure buildup is observed, while in the rectangular case this pressure is easily relaxed by the global deformation, as shown in Figure 10. (**b**) Photo of a ubiquitous void in a cylindrical geometry (circular cross-section) of a cold runner. A spherical bubble-like structure can be seen in the center of the cross-section. Elongated cylindrical voids can form as well.

The pressure change in the molten core, Δp in Equation (1), is integrated from the beginning of the cooling phase, and can reach considerable negative values depending on the shape of the domain (Figure 15a). A sufficiently strong negative pressure can lead to the evaporation of a component within the plastic material, creating a molding defect known as a void [56] (Figure 15b). Vaporization increases the volume of the molten core and decreases the magnitude of the pressure change, which reduces the associated sink mark. The onset of voids depends on the vapour pressure of a particular plastic grade using particular additives and the type of preprocessing, including drying, which determines the moisture content.

### 3.2. Showcase

**Figure 16:**
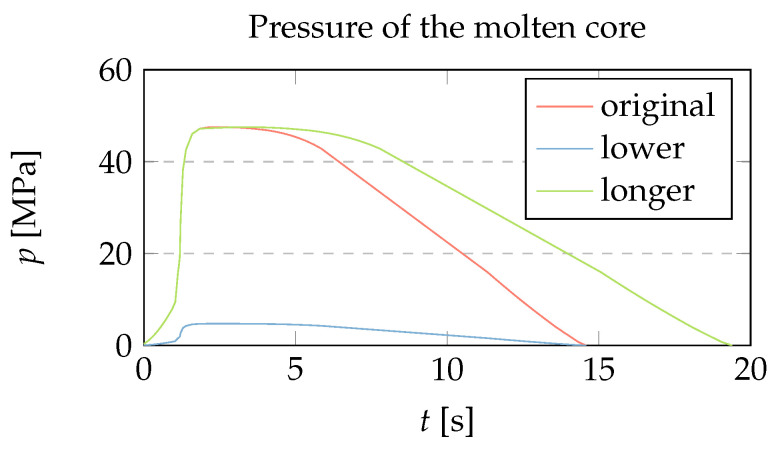
Three variants of the time-dependent pressure of the molten core used to calculate the deformations in Figure 17.

**Figure 17:**
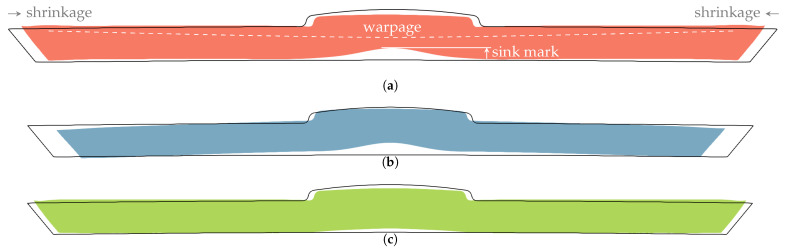
The deformations corresponding to the three pressure traces of Figure 16, scaled by a factor of 20. (**a**) Original deformation of the part, decomposed into global warpage, global length shrinkage and local sink mark; (**b**) A lower packing pressure of the same duration affects global warpage and increases shrinkage, but leaves the depth of the sink mark unchanged; (**c**) A longer packing pressure causes a reduction of the sink mark and global shrinkage, but leaves global warpage largely unchanged.

To support the central claim that shrinkage, global warpage, and local deformations of a part have the same origin and can only be distinguished by characteristic length, we have calculated the influence of three different pressure traces, shown in Figure 16, on the deformation of the part cross-section in Figure 17. The original pressure trace corresponds to the 50 MPa case in Section 3.4 and serves as a reference; the other two are generated synthetically, and are generally achievable through appropriate machine settings, part design, and mold construction. The lower pressure curve has the same shape as the original, except with the values reduced by a factor of 10. The longer pressure curve is obtained by multiplying the time axis after the filling phase by 1.33.

By separating the warpage and the sink mark in Figure 17 on the basis of their different length scales and comparing the magnitudes of all three deformation components in Figure 17a–c, it can be seen that all three components can be controlled by a single process parameter, namely, the time-dependent pressure of the molten core p(t). In addition, it can be seen that the depth of the sink mark is mainly determined by the time at which the pressure drops to zero, while warpage and shrinkage are more influenced by the pressure conditions in the filling and packing phases. The empirical fact that sink marks and warpage can be controlled almost separately by the height and length of the time-dependent pressure is an additional advantage.

### 3.3. Difference between Liquid and Rubbery Core

**Figure 18:**
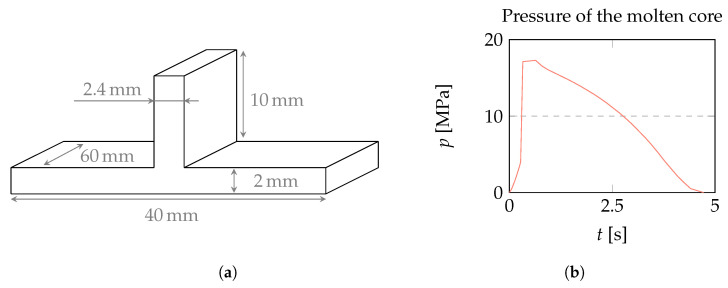
(**a**) Geometry used by Naka et al. [31] and (**b**) time-dependent pressure of the molten core simulated with estimated runner system.

Naka et al. [31] provided a profilometer measurement of the sink mark under the rib for the specific case of a 2 mm-thick base plate with a 2.4 mm-thick rib molded from acrylonitrile butadiene styrene (ABS) amorphous plastic with packing pressure of 20 MPa. We replicated the part geometry in Figure 18a, estimated the feeding system from the sketches, and simulated the injection molding process with Autodesk Moldflow using the molding parameters provided by Naka to obtain the time-dependent pressure p(t) of the molten core in Figure 18b, which is a necessary input for our method.

**Figure 19:**
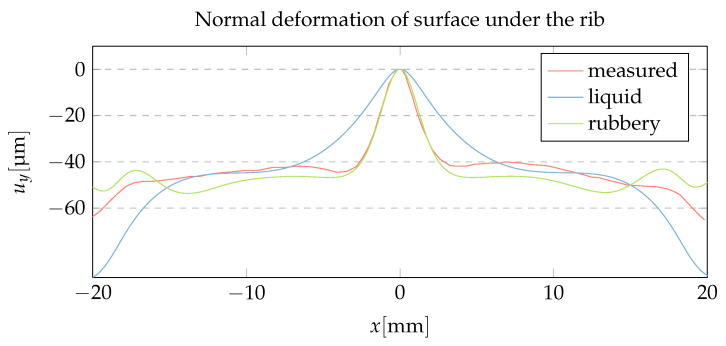
The experiment of Naka [31] simulated with our thermoelastic method: direct comparison of the normal deformation of the surface under the rib with the profilometer measurement of [31].

The displacement of the central cross-section was calculated by treating the molten core as a liquid and as a rubbery material. A comparison of the two approaches with Naka’s profilometer measurement is shown in Figure 19. It can be seen that the liquid approach overestimates the width of the sink mark, while the rubbery approach provides the correct depth and width of the sink mark.

If the pressure persists until the molten core is small and localized below the rib, the liquid and rubbery approaches provide similar results. However, if the pressure drops to zero early when the molten core extends over most of the part, the rubbery approach, with its finite shear modulus, prevents the thermal shrinkage load from acting on the entire molten core, instead localizing its effect and producing narrower sink marks compared to the liquid approach.

### 3.4. Validation

**Figure 20:**
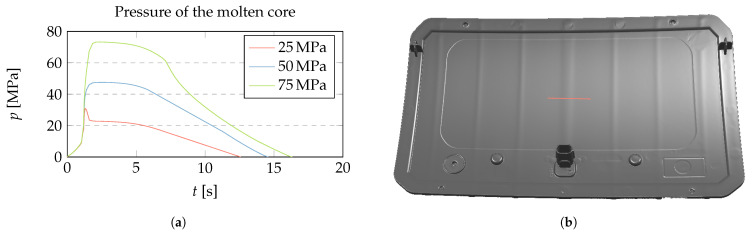
(**a**) Time-dependent pressure of the molten core for three different packing pressure settings simulated with Autodesk Moldflow. (**b**) Render of 3D scan data of a molded part with marked section of investigation. The illumination direction is selected to emphasise surface deformation.

Experimental samples were molded from amorphous polycarbonate (PC) plastic Makrolon 2405 with three constant packing pressure settings of 25 MPa, 50 MPa, and 75 MPa maintained for 6 s. The valve gates were closed before the end of packing to prevent flowback. All other parameters were set according to the material supplier’s recommendations, and the injection rate was set to achieve a 1 s filling time per 100 mm flow length. The produced samples were 3D scanned, and the deformation in the direction of the surface normal was extracted on the path shown in Figure 20b.

**Figure 21:**
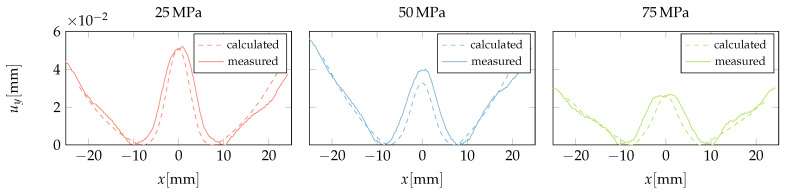
Comparison between surface deformation of molded samples and numerical prediction.

The molding process was simulated with Autodesk Moldflow software to calculate the time-dependent pressure p(t) at the investigated cross section (Figure 20a). The comparison between the surface deformation of the molded samples and its numerical prediction within the liquid core approach is shown in Figure 21. An alignment between the 3D-scanned and nominal shapes is carried out to ensure that the extracted surface deformation was symmetric and zero at the lowest point. In addition, a small parabolic displacement is added to the calculated results to match the measured global warpage, while the shape of the sink mark is left unchanged.

We can conclude that the agreement between the calculated and measured data is good. Due to the long packing phase, the molten core is localized under the rib, which makes the liquid approach suitable.

## 4. Discussion

In this study, we have introduced and applied a method to predict the deformation of injection molded parts. Our approach advances current research on sink mark formation by incorporating both the filling and packing phases of the injection molding process. We establish that global part warpage and localized deformations such as sink marks are due to the same cause, namely, thermal shrinkage and local pressure within the molten core.

By adopting a dual perspective of the molten core, treating it as both a liquid and a rubbery material according to previous studies [36,37,38], we demonstrate that the latter is generally essential for accurate prediction of deformations. On the other hand, the liquid approach can provide correct results if the packing phase is sufficiently long that the molten core can localize when the pressure drops to zero, i.e., at the beginning of the cooling phase.

Although the limitations of the liquid approach are evident, it remains a valuable conceptual tool. It naturally describes the buildup of negative pressure in the molten core that leads to the formation of voids. Furthermore, by studying the outcomes of this simulation model, practitioners can develop an intuitive understanding of where sink mark risks might be apparent when examining the cross-sectional shape of the molded part. This understanding is aided by the identification of stiffer and weaker regions of the solidified shell in relation to the negative pressure load of the molten core.

Looking ahead, several refinements could be incorporated into the thermoelastic method, including:

- Viscoelastic relaxation: with this modification, the value of elastic parameters could be adjusted locally based on the material history. Material parameters for such an approach are increasingly common.
- Variable heat transfer coefficient: during the cooling phase, the heat transfer coefficient could be updated at each time step based on the local distance between the plastic and the mold.
- Evaluation of local detachment: the condition for detachment from the mold walls could be evaluated locally, implementing the boundary conditions for the displacement accordingly. The numerical stability of such an approach remains an open question for further investigation.

The true utility of this method lies not in its theoretical demonstration but in its potential for practical implementation in commercial software, which would make it accessible to the plastic design community. Advances in computing power have made such an implementation feasible within existing three-dimensional simulation procedures. Warpage calculation of predefined critical areas with a limited number of discretization points could be improved with such a method. Future validation procedures would reveal the extent to which the filling and packing phases are already adequately addressed within current procedures and whether the primary benefit of this method does indeed lie in its capability to incrementally calculate the displacement of solidified plastic during the cooling phase.

## Figures and Tables

**Figure A1:**
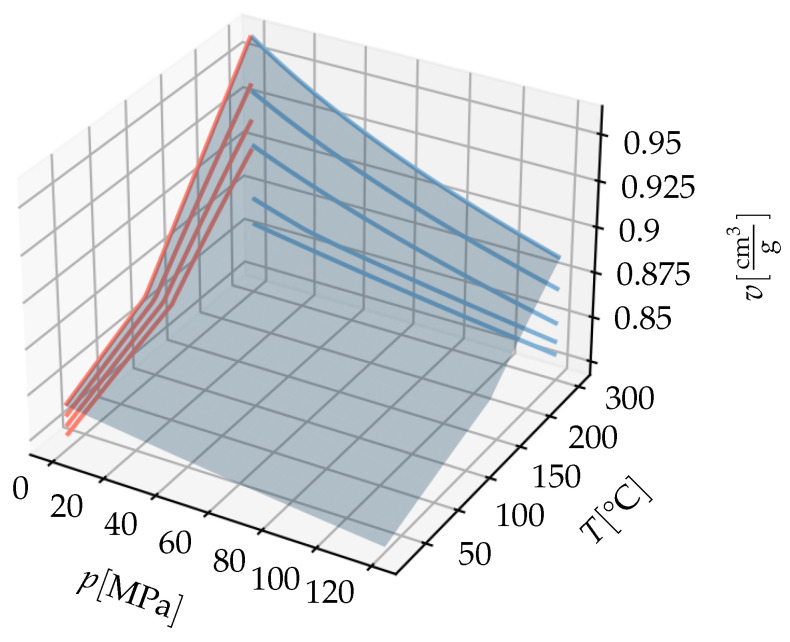
pvT diagram with projected contours.

**Figure A2:**
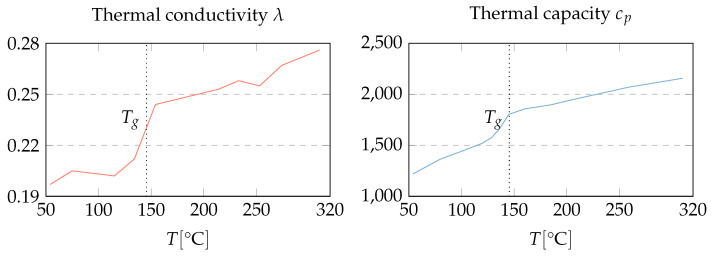
Temperature-dependent thermal properties from the material characterization report of PC Makrolon 2405. Courtesy of Covestro Deutschland AG.

## Appendix A. Modeling the Equation of State

The relationship between pressure, volume and temperature (pvT) is described by an equation of state. A commonly used model for the equation of state in plastic materials is the two-domain Tait equation [57,58], as it accurately represents the measured [59] pvT behavior of amorphous plastic materials. This particular equation separates the pvT space into two distinct regions, which are temperature-dependent: the melt region (*m*) above the glass transition temperature $T_{g}$, and the solid region (*s*) below it

(A1) $$T_{g}\left(p\right)=b_{5}+b_{6}p.$$

Specific volume-temperature effects in each region are captured by

(A2) $$v_{0m}=b_{1m}+b_{2m}\left(T-b_{5}\right),\;v_{0s}=b_{1s}+b_{2s}\left(T-b_{5}\right),$$

while the effect of pressure is provided by

(A3) $$\;B\left(T\right)=b_{3m}\exp\left[-b_{4m}\left(T-b_{5}\right)\right],\;B\left(T\right)=b_{3s}\exp\left[-b_{4s}\left(T-b_{5}\right)\right],$$

which is combined in the Tait equation:

(A4) $$v\left(T,p\right)=v_{0}\left(T\right)\left[1-C\log\left(1+\frac{p}{B(T)}\right)\right].$$

To capture the pvT behavior of semi-crystalline materials, an additional term

(A5) $$v_{t}=b_{7}\exp\left[b_{8}\left(T-b_{5}\right)-b_{9}p\right]$$

is added to the expression (A4). While this term increases the accuracy of the Tait model, it does not incorporate the complex evolution of crystallinity, which generally must be modeled explicitly. Several studies [21,60] have used calorimetry measurements to determine the cooling rate-dependent volume. Zhou et al. [61] proposed a comprehensive two-scale model using the Monte Carlo method to model the evolution of crystallinity and crystal morphology. To limit the scope of this work, only sink marks on parts molded with amorphous plastic materials PC and ABS were studied.

**Table A1:** Two-domain Tait model parameters for PC Makrolon 2405.

Parameter	Value	Unit
$b_{1m}$	8.649 × 10^−4^	m${}^{3}$kg${}^{-1}$
$b_{2m}$	6.028 × 10^−7^	$m^{3}kg{}^{-1}K{}^{-1}$
$b_{3m}$	1.6934 × 10^8^	Pa
$b_{4m}$	4.343 × 10^−3^	$K{}^{-1}$
$b_{1s}$	8.649 × 10^−4^	$m^{3}kg{}^{-1}$
$b_{2s}$	2.034 × 10^−7^	$m^{3}kg{}^{-1}K{}^{-1}$
$b_{3s}$	2.76286 × 10^8^	Pa
$b_{4s}$	2.373 × 10^−3^	$K{}^{-1}$
$b_{5}$	4.1217 × 10^2^	K
$b_{6}$	4.239 × 10^−7^	$\mathrm{KPa}{}^{-1}$

Courtesy of Covestro Deutschland AG.

## Appendix B. Modeling the Thermal Properties

To solve the heat equation, the thermal conductivity λ and heat capacity $c_{p}$ must be known. Generally, λ is a function of temperature, as shown in Figure A2. In the past, commercial software relied on a single value for λ; however, measurements over a wide range of temperatures are now increasingly common. Nevertheless, the relative change of λ over the relevant temperature range is typically small, which means that the use of a single value did not significantly compromise the accuracy of the simulations. Heat capacity is usually an increasing function of temperature. Figure A2 presents the temperature-dependent heat capacity for the same plastic material. Accurate representation of these temperature-dependent properties is essential for precise prediction of the temperature field and cooling behavior in injection molding simulations.

## Appendix C. Modeling the Elastic Properties

The mechanical behavior of materials in linear elastic approximation is modeled by Hooke’s law (Equation (6)), which for isotropic materials requires two elastic constants. We chose the bulk modulus *K*, which can be directly calculated from the equation of state

(A6) $$\frac{1}{K}=-\frac{1}{v}\frac{\partial v}{\partial p},$$

and Young’s modulus *E* of uniaxial deformation. This modulus is measured at room temperature.

(A7) $$E_{s}=2400\;\mathrm{MPa}$$

The datawere provided by Covestro AG. The Young’s modulus of the rubbery state is three orders of magnitude lower [36,48,49]:

(A8) $$E_{r}=2.4\;\mathrm{MPa}.$$

A narrow transition region is employed to interpolate between $E_{s}$ and $E_{r}$.

A third constant is needed to link temperature change to deformation. The coefficient of thermal expansion (CTE) is well known [54] and can be calculated from the equation of state:

(A9) $$\alpha =\frac{1}{v}\frac{\partial v}{\partial T}.$$

The material constants are shown in Figure A3.
